# Subterranean biodiversity and depth distribution of myriapods in forested scree slopes of Central Europe

**DOI:** 10.3897/zookeys.930.48914

**Published:** 2020-04-28

**Authors:** Beáta Haľková, Ivan Hadrián Tuf, Karel Tajovský, Andrej Mock

**Affiliations:** 1 Institute of Biology and Ecology, Faculty of Science, Pavol Jozef Šafárik University, Košice, Slovakia Pavol Jozef Šafárik University Košice Slovakia; 2 Department of Ecology and Environmental Sciences, Faculty of Science, Palacky University, Olomouc, Czech Republic Palacky University Olomouc Czech Republic; 3 Institute of Soil Biology, Biology Centre CAS, České Budějovice, Czech Republic Institute of Soil Biology, Biology Centre CAS České Budějovice Czech Republic

**Keywords:** Chilopoda, Diplopoda, Myriapoda, MSS, subterranean traps

## Abstract

The shallow underground of rock debris is a unique animal refuge. Nevertheless, the research of this habitat lags far behind the study of caves and soil, due to technical and time-consuming demands. Data on Myriapoda in scree habitat from eleven localities in seven different geomorphological units of the Czech and Slovak Republics were processed. Based on previous studies, as well as knowledge of cave and soil fauna, it was hypothesised that the occurrence of a varied and peculiar fauna would show a pattern of depth distribution with variations due to local specificities. From 2005–2016 (at least one year on each site), macrofauna was collected via sets of three long-term exposed subterranean traps consisting of 110 cm long perforated tube, with ten cups located in a gradient at 5–95 cm below the soil surface. In total, 14 symphylans (not identified to species level), 271 centipedes (23 spp.) and 572 millipedes (32 spp.) were sampled. The overall depth distribution of centipedes and millipedes appeared to have relatively similar pattern, with both groups being found at all depth levels. Nevertheless, this pattern depends on locations. The depth distribution trend lines are mostly in the form of an asymmetric ‘U’, with decreased abundance until the middle of the gradient, followed by increase in the deepest levels. Epigeic species were sporadically distributed along the whole depth gradient, but concentrated at the soil surface, while some subterranean species, such as the centipede *Lithobius lucifugus* and the millipedes *Geoglomeris subterranea*, *Cibiniulus slovacus* and *Archiboreoiulus pallidus*, were recorded in the deepest parts of the gradient. This characterises the debris community as a mixture of soil and subterranean species with an absence of species exclusively found in caves. The use of different fixation methods in traps had a significant and selective impact on samples; millipedes were either attracted by ethylene glycol or repelled by formaldehyde. Centipedes were also captured more frequently in ethylene glycol; however, the species composition varied in each of the fixatives. Depth distribution of myriapods was similar in both fixative solutions. Traps with these fixatives could be recommended for similar ecological studies.

*The paper is dedicated to Christian Juberthie (12 Mar 1931–7 Nov 2019), the author of the concept of MSS (milieu souterrain superficiel) and the doyen of modern biospeleology*

## Introduction

Forested scree slopes (slope deposits) represent a unique type of shallow subterranean domain, which are frequently labelled in literature as shallow subterranean habitat (SSH; Culver and Pipan 2014) or meso-void shallow substratum (milieu souterrain superficiel, MSS of Juberthie et al. 1980; preferred abbreviation for this paper). The main condition for the existence of MSS is the presence of stone deposits forming in the underground environment. Weathered rock fragments and sediments accumulate in several layers, which are subsequently covered by the soil. Due to this isolation from the surface, MSS serves as a stable habitat with conditions similar to those in caves (Juberthie 2000; Giachino and Vailati 2010). Cracks and air-filled voids create a network of interconnected corridors that provide suitable shelter for various groups of invertebrates, but also serve as a refugium for relic fauna (Růžička and Klimeš 2005; Zacharda et al. 2005; Mock et al. 2015; Kováč et al. 2016; Růžička et al. 2016; Nitzu 2016; Tuf et al. 2017; Nitzu et al. 2018b; Mammola 2018). Shallow subterranean habitats can be defined as a habitat on a transition zone with the ongoing adaptation of organisms to underground life (Pipan et al. 2011; Ortuño et al. 2013; Nitzu et al. 2014; Mammola et al. 2017). The presence of invertebrate life forms possessing convergent morphological adaptations (depigmentation, eye absence, reduction of size, etc.) to subterranean life is one of the main criteria for distinguishing surface from underground habitats.

The study of deeper layers of forested scree slopes reveals that refugia of rare fauna add another dimension of environmental heterogeneity affecting overall biodiversity. Rather numerous studies on the ecology of various groups of fauna in MSS in Europe were conducted (reviewed in Mammola et al. 2018), some of them focusing on scree slopes of Slovakia and the Czech Republic (Růžička and Klimeš 2005; Laška et al. 2011; Rendoš et al. 2012, 2014, 2016a; Mock et al. 2015; Růžička and Dolanský 2016; Rudy et al. 2018; Jakšová et al. 2019; Jureková et al. 2019). Subterranean diversity of Myriapoda inhabiting underground of scree slopes has been investigated as well (Nitzu et al. 1999; Ilie 2003a, 2003b; Rendoš et al. 2012, 2016a; Jiménez-Valverde et al. 2015; Mock et al. 2015; Tuf et al. 2017); some studies even have described new myriapod taxa (e.g., Stoev 2008; Gilgado et al. 2015; Akkari et al. 2018). Attention should also be paid to the results of studies testing the reliability of the use of a set of subterranean traps inserted to the substrate through plastic pipe and other methodological aspects. The type of fixative solution and trap exposure duration can also affect species assemblages (Mammola et al. 2016; Jureková et al. 2019). Additionally, environmental variables affect the distribution pattern of invertebrates below the surface of stone debris (Rendoš et al. 2016b; Nitzu et al. 2018a). Our contribution completes the knowledge from a number of little or unstudied sites of Slovak and the Czech republics. The aims of our study were to (1) analyse the structure and diversity of myriapods dwelling in forested scree slopes, and (2) to generalise the distribution of myriapods along the depth gradient at selected locations in Central Europe.

## Materials and methods

### Study sites

**Figure 1.**
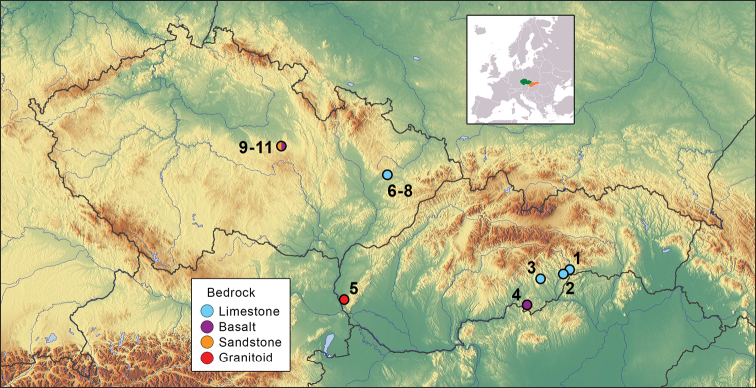
Location of the study sites. **1** Doline next to Silická ľadnica Ice Cave **2** Vysoká Hill (both sites in Slovak Karst National Park) **3** Drienok Valley (Revúcka Highlands) **4** Belinské skaly (Cerová vrchovina Highlands) **5** Okopanec Hill (Malé Karpaty Mts.) **6–8** Three localities near the Zbrašov Aragonite Caves and Hůrka u Hranic (Moravian-Silesian Foothills) **9–11** Three localities in Chrudim region (Iron Mts.).

The study was carried out from 2005 to 2016 at various locations situated in different geomorphological units of Slovakia and the Czech Republic. Five of the forested scree slopes were situated in four geomorphological units in Slovakia; six study sites were part of two geomorphological units in the Czech Republic (Fig. 1 and Table 1).

**Table 1.** Characteristics of the scree slopes study sites. The numbers indicating particular study are presented in Fig. 1. Key: Alt. = altitude, Expo. days = trap exposure time.

Study site	Coordinates	Alt. (m)	Bedrock	Slope aspect	Sampling period	Expo. days
**1**	48°33'N, 20°30'E	489	Limestone	W	11 Jun 2014–29 Apr 2015	322
**2**	48°31'N, 20°25'E	328	Limestone	SW	11 Jun 2014–29 Apr 2015	322
**3**	48°32'N, 20°07'E	315	Limestone	N	15 May 2012–17 Oct 2013	520
**4**	48°13'N, 19°52'E	460	Basalt	SW	15 May 2012–17 Oct 2013	520
**5**	49°77'N, 17°66'E	410	Granitoid	SW	15 Jan 2015–16 Jan 2016	365
**6**	49°31'N, 17°44'E	325	Limestone	E	1 Feb 2005–1 Mar 2006	393
**7**	49°32'N, 17°44'E	375	Limestone	W	1 Feb 2005–1 Mar 2006	393
**8**	49°32'N, 17°44'E	375	Limestone	W	1 Feb 2005–1 Mar 2006	393
**9**	49°50'N, 16°04'E	460	Sandstone	SE	7 Mar 2005–24 Mar 2006	382
**10**	49°49'N, 16°02'E	400	Sandstone	NW	7 Mar 2005–24 Mar 2006	382
**11**	49°50'N, 16°02'E	455	Basalt	W	7 Mar 2005–24 Mar 2006	382

1. Forested scree slope in the karst doline (sinkhole) were close to the collapse entrance of the Silická ľadnica Ice Cave, Slovak Karst National Park (site 1; Fig. 1). Situated on limestone bedrock, the site is characterised by dense vegetative cover, forested by linden-hornbeam and maple trees (*Tilio*-*Aceretum* with *Carpinus betulus*, *Urtica* sp., *Lunaria* sp., *Galium* sp. in herb layer). Scree profile is divided to leaf litter and humus (0–10 cm), organo-mineral layer (10–30 cm) and rock fragments layer (30–110 cm).

2. Forested scree slope of the Vysoká Hill is situated approximately 30 m from the entrance of Ardovská jaskyňa Cave, Slovak Karst National Park (site 2; Fig. 1). Situated on limestone bedrock, the vegetative cover on the slope is poor, with the presence of dogwood-maple trees (*Corno*-*Carpinetum*) and bryophytes. Scree profile is characterised by a layer of leaf litter and humus (0–15 cm), organo-mineral layer mixed with tiny rocks (15–75 cm) and a large rock layer (75–110 cm).

3. Northern limestone scree slope in the Drienok Valley is located a few meters below the entrance to the Špaňopoľská Cave, Revúcka vrchovina Highlands (site 3; Fig. 1). The slope is overgrown with beech-hornbeam forest (*Fago*-*Caripetum*) and the scree slope profile is characterised by a layer of leaf litter and humus (0–5 cm), organo-mineral layer (5–25 cm), a layer of a mixture of soil and rocks (25–70 cm) and layer of weathered rock fragments (70–110 cm).

4. Southwestern scree slope is in the Belinské skaly National Nature Monument, Cerová vrchovina Highlands (site 4; Fig. 1). Situated on basalt bedrock, the slope is covered with xerophilous oak-hornbeam trees (*Querco*-*Carpinetum*). Scree profile consists of litter and humus (0–5 cm), organo-mineral layer (5–30 cm) and scree with mineralised soils (30–110 cm).

5. Forested south scree slope is on the Okopanec Hill, Malé Karpaty Mountains (site 5; Fig. 1). The granitoid slope characterised by poor vegetative cover with the presence of beech trees and bryophytes. Scree slope profile consists of leaf litter and humus (0–5 cm), organo-mineral layer (5–20 cm), a layer of rock segments and mineralised soil (20–75) and scree partially filled with soil and tree roots (75–110 cm).

6. Limestone scree slope is a part of the Zbrašov Aragonite Caves National Natural Reserve, Moravian-Silesian Foothills (site 6; Fig. 1). The site is situated 50 m from the administration building. Vegetative cover is represented by deciduous trees, predominantly *Acer* sp., *Fraxinus excelsior* and *Tilia* sp. with occasional occurrence of *Quercus* sp. and *Robinia pseudacacia*. Soil profile consists of scree made up of stone fragments ranging from a few centimetres in diameter to larger rocks. The interspace between the limestone rock fragments is filled with humus formed by leaf litter decomposition.

7. Limestone scree slope is located above the right bank of the Bečva River, southern part of the Hůrka u Hranic National Natural Reserve, Moravian-Silesian Foothills (site 7; Fig. 1). The slope is overgrown with old trees of *Fagus sylvatica* and *Quercus* sp.; occasionally *Carpinus betulus*, *Acer* sp., and *Fraxinus excelsior* are present. Due to close proximity to the limestone wall, the soil contains many small limestone fragments of various sizes with interspace filled with yellow clay.

8. Limestone scree slope is located above the right edge of the Bečva River, southern part of the Hůrka u Hranic National Natural Reserve, Moravian-Silesian Foothills (site 8; Fig. 1); this site is close to study site 7, but further away from the limestone wall. Vegetative cover is the same as at study site 7. The size of the debris particles ranges from a few centimetres to tens of centimetres. Scree profile is formed by larger fragments on the surface, the spaces between them filled with humus; the lowest layer consists of smaller fragment, with spaces filled with yellow clay.

9. Sandstone scree slope is at the edge of a beech forest, cadastral area of the Hluboká village, Chrudim region, Železné hory (Iron Mountains; site 9; Fig. 1). Vegetative cover consists predominantly of *Trifolium pratense*, *Impatiens noli-tangere*, *Impatiens parviflora*, *Anemone nemorosa*, *Convallaria majalis*, *Asperula odorata*, and *Polygonatum multiflorum*. The greatest relative abundance of the tree community is represented by *Fagus sylvatica*, with the presence of *Abies alba* and *Picea abies*. The scree profile is characterised by beech leaf litter with undecomposed leaves at the surface (10 cm), sandy soil (20 cm) and a layer of clay (> 20 cm).

10. Basalt scree slope is in the vicinity of the Hněvětice village, Chrudim region, Železné hory (Iron Mountains; site 10; Fig. 1). The average stone fragment size is about 10 cm in diameter. The slope is overgrown with mixed forest, predominantly *Betula pendula*, *Carpinus betulus*, *Picea abies*, and *Fagus sylvatica*. The scree was covered with a thin layer of undecomposed leaves on the surface. The soil was homogeneous throughout the whole depth gradient. Stone fragments were few centimetres in diameter. The spaces between the stones were filled clay.

11. Scree slope was situated next to a former basalt quarry in the cadastral area of the Hněvětice village, Chrudim region, Železné hory (Iron Mountains; site 11; Fig. 1). The area is treeless. Poor vegetative cover is represented mainly by *Tussilago farfara* and *Crepis biennis*, with the presence of some ruderal weeds (e.g., *Atriplex patula*, *Rumex acetosa*, *Rumex obtusifolius*). Soil on the study site was almost homogeneous to the depth of 60 cm and consisted of clay with a large amount of stone fragments. With increasing depth, the presence of clay particles intensified.

### Sampling

To capture myriapods, subterranean pitfall traps were used, constructed by Schlick-Steiner and Schlick. These traps were finely modified according to the available construction material and there was little difference between those used in the Czech and Slovak study sites. These differences should not have affected the monitored parameters of the myriapod communities.

At study sites 1–5, the traps consisted of 110 cm long PVC tube, 10 cm in diameter, perforated at 10 horizontal levels (5, 15, 25…95 cm), circumferentially. Perforations were 0.7 cm in diameter and served as an entrance to the traps for studied fauna. Inside the plastic tube, ten plastic cups (volume 500 ml) were inserted, connected with threated rod (forming 10 cm spacing between each cup) and aligned directly under the perforations to allow animals to be trapped at particular level. We used 4% formaldehyde a fixative solution in two traps at each site and either 50% ethylene glycol in one trap at study sites 1–4 or 11% ethylene glycol in one trap at study site 5. Traps at study sites 6–11 were constructed according to the same design, with a few differences. Instead of drilled perforations at ten levels, three transverse cuts were made in the plastic tube at each of the depth levels, so that three pillars remained in between the cuts to keep the tube together. The cuts were 0.4 cm wide and 9 cm long and served as an entrance for animals to the traps. Inside the plastic cups, only 4% formaldehyde was used as a fixative. All fixatives were diluted in water to the appropriate concentrations.

At each of the study sites, we installed a set of three traps, 1–2 m apart. The plastic pipes with the traps were inserted in a horizontal line into a dug longitudinal pit. The excavated substrate was returned to the pit roughly in layers as it was dug. Approximately 1 month after placing traps into the substrate, they were controlled, and the fixation solution was replaced to avoid the effect of mixing the substrate when digging pits for deep invertebrate distribution.

Subterranean traps were exposed for approximately one year at each of the 11 study sites and controlled regularly. Sampling intervals varied for each study site (Table 1). The plastic cups were pulled out to retrieve the sampled specimens; the content was collected and transported to laboratory. Myriapods were fixed in ethyl alcohol and identified to species level.

### Community characteristics and data analysis

In order to describe myriapod communities, we calculated dominance (D), constancy (C), Shannon’s diversity index (*H*’), and Pielou’s evenness index (*J*’) for centipedes and millipedes separately; indices were estimated separately for each of the study sites. In addition, *H*’ was calculated for each depth of the gradient, for centipedes and millipedes separately. Patterns of depth distribution in overall material of millipedes and centipedes were tested using fitted Generalised Additive Models (GAM) in Canoco 5.0 program. Distributions of species with more than two trapped specimens were tested; only species with significant pattern of its distribution were illustrated in figures.

## Results

Myriapoda were recorded at all eleven study sites and were one of the less frequent groups of arthropods. In total, 857 individuals were identified to 55 species. Diplopoda, unlike to ever-present Chilopoda, were missing at study site 11; however, it was the richest represented class of myriapods, regarding both individuals and species. Symphyla were represented only by 14 individuals at four study sites. The fourth myriapod class, Pauropoda, was not documented at any of the study sites. Species diversity and depth distribution showed geographical differences, but overall, two dominant groups demonstrated similar indicators. Alternation of two fixative solutions in traps resulted in significant differences in both the number of individuals and the species composition, with Diplopoda and Chilopoda responding differently to the type of fixation.

Representatives of Symphyla were captured at four of the study sites. All collected individuals (not identified to species level) were distributed unevenly along the depth gradient, present at almost every depth of the top half of the gradient. One third of all captured symphylans were present in the bottom half of the gradient, at depths of 65 and 95 cm. Almost two-thirds of Symphyla specimens were captured in traps with formaldehyde.

### Centipedes

**Figure 2.**
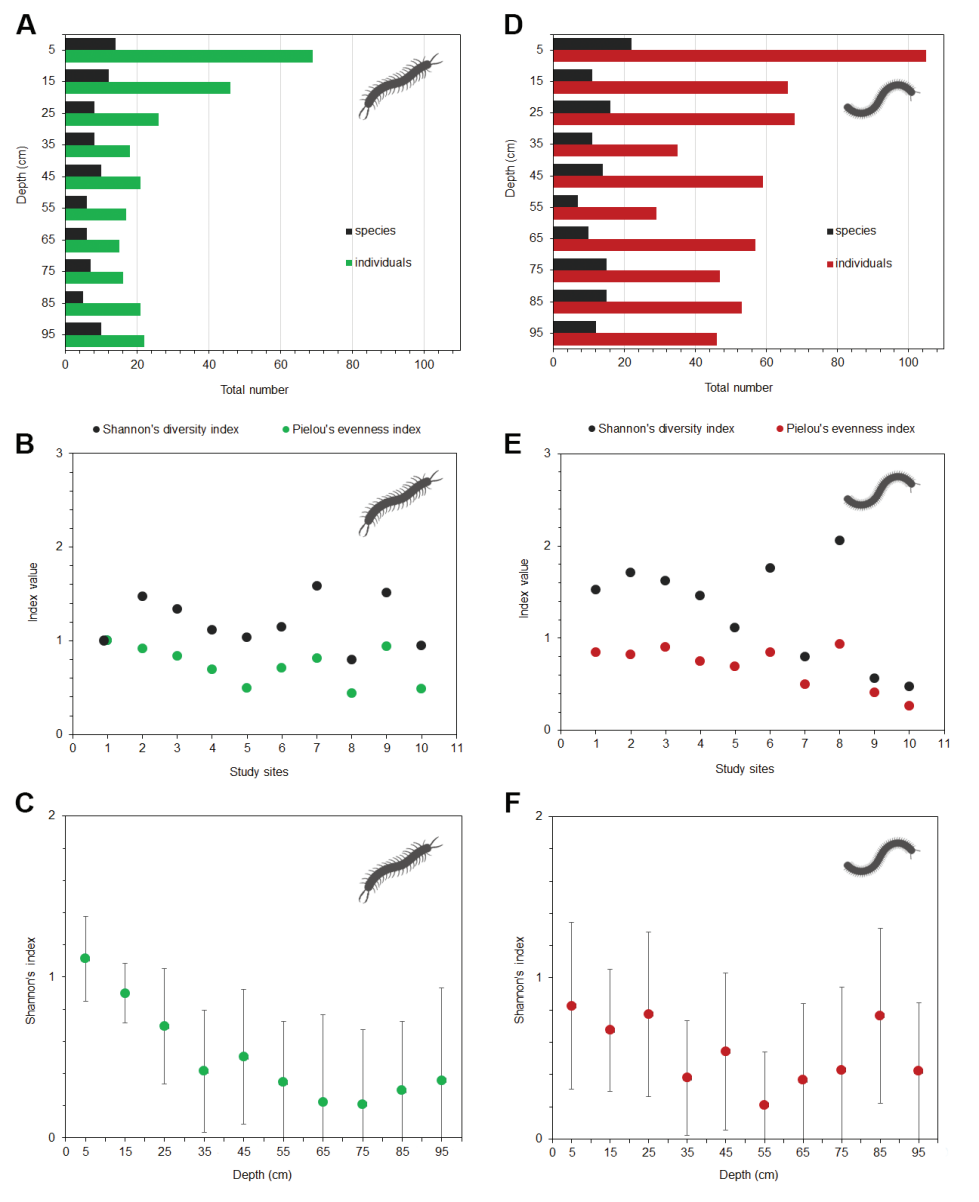
**A** Overall depth distribution of centipede individuals and species **B** values of Shannon’s diversity index and Pielou’s evenness index, calculated for centipedes, at each of the study sites **C** mean values of Shannon’s diversity index (±SD) calculated for centipedes, at each depth of the gradient (summarised data from all localities) **D** overall depth distribution of millipede individuals and species **E** values of Shannon’s diversity index and Pielou’s evenness index, calculated for millipedes, at each of the study sites **F** mean values of Shannon’s diversity index (±SD) calculated for millipedes, at each depth of the gradient.

Overall, 271 specimens of Chilopoda were sampled, belonging to 23 species and five families (See Suppl. material 1: Table S1). The number of centipede species sampled on individual study sites varied from 1–8 species, with an average of 5.2 species sampled per site. The highest value of Shannon’s diversity index (*H*’ = 1.58) was recorded on the scree slope at the edge of the Bečva River (site 7), while the highest value of Pielou’s evenness index (*J*’ = 1) was recorded at the scree slope near Silická ľadnica Ice Cave (site 1) (Fig. 2B). At the scree slope next to the quarry of the village of Hněvětice (site 11), only one species, *Lamyctes emarginatus*, was caught in the traps. Among collected Chilopoda, two species can be classified as eudominant, *Lithobius forficatus* (D = 47%) and *Lithobius lucifugus* (D = 14%); *Lithobius forficatus* represented the species with the highest constancy (C = 82%), occurring at nine of eleven sites.

**Figure 3.**
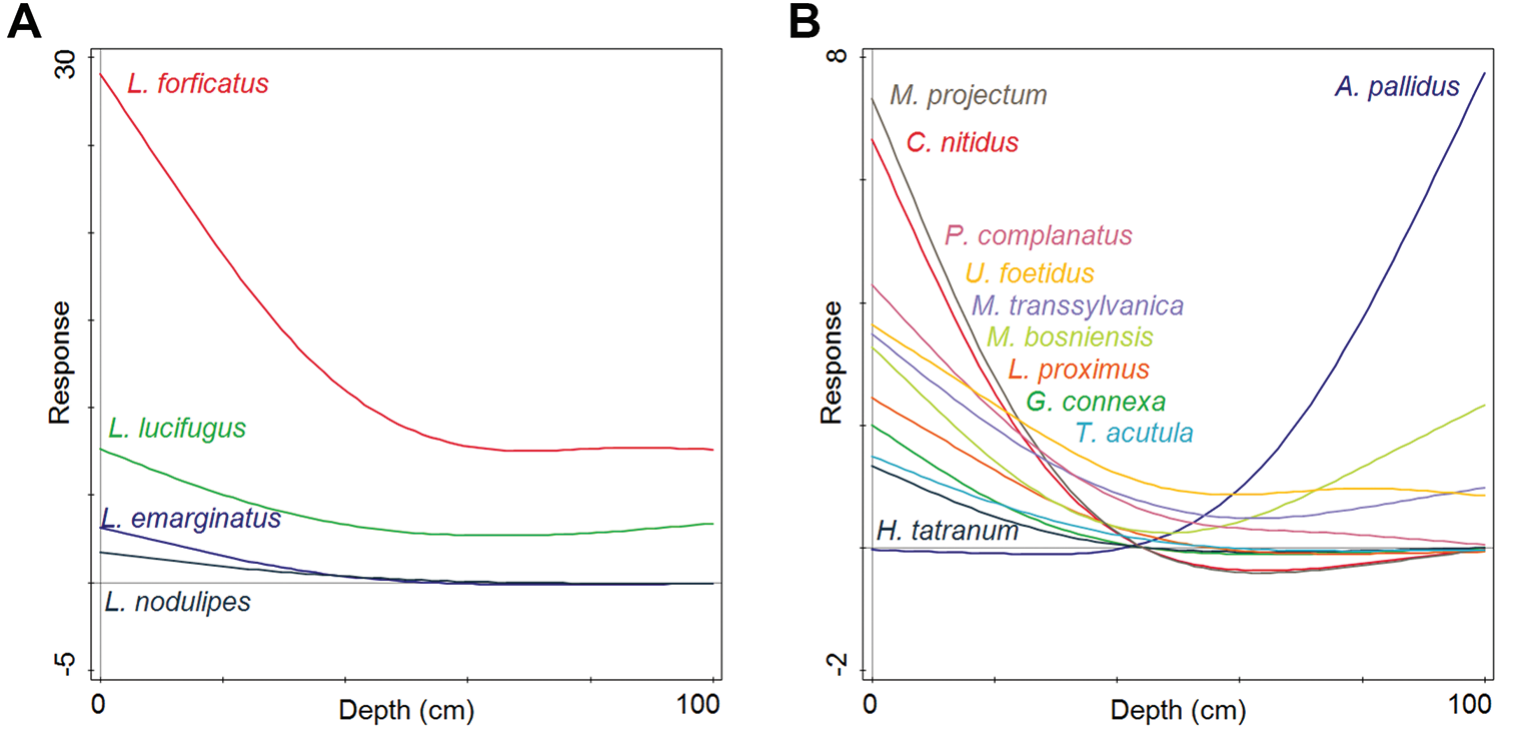
Generalised Additive Models of depth distribution pattern of **A** centipedes and **B** millipedes. Only species with significant pattern are illustrated. (F-values, * *p* < 0.05, ** *p* < 0.01): **A***Lamyctes emarginatus* (13.1**), *Lithobius forficatus* (17.2**), *Lithobius lucifugus* (5.0*), *Lithobius nodulipes* (9.7**) **B***Archiboreoiulus pallidus* (22.7**), *Cylindroiulus nitidus* (5.4*), *Glomeris connexa* (5.4*), *Hylebainosoma tatranum* (5.4*), *Leptoiulus proximus* (7.3*), *Mastigona bosniensis* (10.5**), *Megaphyllum projectum* (5.4*), *Melogona transsylvanica* (15.2**), *Polydesmus complanatus* (13.1**), *Trachysphaera acutula* (5.4*), *Unciger foetidus* (4.9*).

Regarding depth distribution of centipedes, the highest numbers of individuals and species were captured near the surface; however, the overall distribution curve resembled U-shape with decreasing abundances in upper part of profile replaced by opposite pattern in deepest layers (Fig. 2A). More than half of the species were sampled only in the first half of the depth profile (5–45 cm), while four species were collected exclusively in the uppermost levels (5–15 cm). None of the species were observed solely in the deeper half of the gradient (Table 2). Shannon’s diversity index (*H*’) was highest at a depth of 95 cm; however, at 5 cm depth, the value was really similar (Fig. 2C). *Lithobius forficatus* and *L. lucifugus* were the only two Chilopoda species with an occurrence at all layers of the depth profile; *L. forficatus* showed a significant affinity to the surface (F = 17.2, *p* < 0.01), while none of the species showed significant positive relation to deepest layers of the depth gradient (Fig. 3A).

**Table 2.** A summary overview of the centipede depth distribution in the eleven scree slopes of Slovakia and the Czech Republic.

Depth (cm)	5	15	25	35	45	55	65	75	85	95	Σ
*Clinopodes flavidus*	2	–	–	1	–	–	–	–	–	–	3
*Cryptops parisi*	1	1	–	1	2	2	–	1	2	1	11
*Geophilus electricus*	1	–	–	–	–	–	–	–	–	–	1
*Geophilus insculptus*	–	1	–	–	–	1	–	–	–	–	2
*Geophilus flavus*	1	–	1	1	–	3	1	–	–	–	7
*Harpolithobius anodus*	4	–	–	–	1	–	–	–	–	1	6
*Lamyctes emarginatus*	3	3	–	–	–	–	–	–	–	–	6
*Lithobius agilis*	–	2	–	1	–	1	–	–	–	–	4
*Lithobius austriacus*	–	1	–	–	–	–	–	–	–	1	2
*Lithobius cyrtopus*	–	1	–	–	–	–	–	–	–	–	1
*Lithobius dentatus*	–	–	–	–	1	–	–	–	–	–	1
*Lithobius forficatus*	29	25	14	9	8	8	5	9	14	5	126
*Lithobius lucifugus*	9	4	4	3	2	2	5	1	3	4	37
*Lithobius macilentus*	–	–	–	–	2	–	–	–	–	1	3
*Lithobius microps*	–	–	–	–	1	–	–	–	–	–	1
*Lithobius mutabilis*	2	5	1	–	–	–	1	–	–	–	9
*Lithobius muticus*	6	–	–	–	1	–	–	1	–	–	8
*Lithobius nodulipes*	2	1	–	1	–	–	–	–	–	–	4
*Lithobius tenebrosus*	–	–	–	1	–	–	–	–	–	–	1
*Lithobius t. fennoscandius*	1	–	1	–	1	–	1	2	1	1	8
*Strigamia acuminata*	4	1	1	–	2	–	–	1	–	5	14
*Strigamia crassipes*	–	–	1	–	–	–	–	–	–	1	2
*Strigamia transsilvanica*	4	1	3	–	–	–	2	1	1	2	14
Σ	69	46	26	18	21	17	15	16	21	22	271

### Millipedes

Diplopoda were represented by 572 individuals (including unidentified juveniles), belonging to 32 species and 12 families (see Suppl.material 2: Table S2). The highest number of species (nine species) was recorded at study site 8, with the complete absence of millipedes at one of the study sites (quarry, locality 11). On average, 5.5 species per site were sampled. The highest value of both Shannon’s diversity index and Pielou’s evenness index (*H*’ = 2.06 and *J*’ = 0.94) was recorded on the scree slope at the edge of the Bečva River (site 8; Fig. 2E). The only eudominant species of Diplopoda was *Ochogona caroli* (D = 40%). None of the species occurred on more than half of the study sites, with *Unciger foetidus* representing the species with the highest constancy (C = 46%; occurred at five of eleven sites).

The distribution of millipedes along the depth profile was non-uniform. The highest numbers of individuals and species were sampled at the depth of 5 cm (Fig. 2D). One third of the species were captured solely in the top half of the depth gradient (5–45 cm), with five species sampled exclusively in the uppermost layers (5–15 cm). Shannon’s diversity index was highest at a depth of 5 cm; the value was lowest at 65 cm (Fig. 2F). Three species, *Craspedosoma transsylvanicum*, *Polyzonium germanicum*, and *Trachysphaera costata* occurred solely in the deeper half of the depth gradient (85–95 cm); however, they were represented by low number of individuals (Table 3). *Megaphyllum projectum* and *Cylindroiulus nitidus* showed a significant positive correlation to the surface (both species F = 5.4, *p* < 0.05); other species with affinity for the upper part of the depth gradient are depicted in the left part of the Fig. 3B. The millipedes *Mastigona bosniensis* and *Melogona transsylvanica* were abundant on the surface as well as in deepest layers. The only species significantly more abundant at lower depths was *Archiboreoiulus pallidus* (F = 22.7, *p* < 0.01). Nevertheless, another species of the same morphotype (thin blind julid without pigment), *Cibiniulus slovacus* was also sampled exclusively in deeper zones of scree habitats.

**Table 3.** A summary overview of the millipede depth distribution in the eleven scree slopes of Slovakia and The Czech Republic. Seven juvenile individuals could not be identified to species level.

Depth (cm)	5	15	25	35	45	55	65	75	85	95	Σ
*Archiboreoiulus pallidus*	–	–	1	–	–	–	–	2	4	9	16
*Blaniulus guttulatus*	2	3	2	2	3	–	2	1	3	5	23
*Brachydesmus superus*	–	2	1	–	–	–	1	–	1	–	5
*Cibiniulus slovacus*	–	–	–	–	4	1	–	6	4	–	15
*Craspedosoma transsylvanicum*	–	–	–	–	–	–	–	–	–	1	1
*Cylindroiulus nitidus*	10	–	–	–	–	–	–	–	–	–	10
*Geoglomeris subterranea*	–	–	–	–	1	1	–	–	–	–	2
*Glomeris connexa*	3	–	–	–	–	–	–	–	–	–	3
*Glomeris tetrasticha*	–	–	–	–	2	–	–	–	1	1	4
*Haasea flavescens*	2	12	2	1	1	3	–	1	4	–	26
*Haplogona oculodistincta*	3	8	16	4	4	7	3	–	4	2	51
*Hungarosoma bokori*	1	–	–	–	1	–	–	–	–	–	2
*Hylebainosoma tatranum*	2	–	–	–	–	–	–	–	–	–	2
*Leptoiulus baconyensis*	1	–	–	–	–	–	–	–	–	–	1
*Leptoiulus proximus*	2	3	–	–	–	–	–	–	–	–	5
*Leptoiulus trilobatus*	3	–	–	2	–	1	–	–	–	–	6
*Listrocheritium septentrionale*	5	–	1	2	2	1	2	2	1	1	17
*Mastigona bosniensis*	4	2	–	–	–	–	–	2	2	2	12
*Megaphyllum projectum*	11	–	–	–	–	–	–	–	–	–	11
*Melogona transsylvanica*	4	2	1	1	1	–	–	1	1	1	12
*Melogona voigtii*	2	–	3	2	1	–	–	1	1	–	10
*Ochogona caroli*	21	21	24	18	33	15	41	21	17	18	229
*Ommatoiulus sabulosus*	1	–	–	–	–	–	–	1	–	–	2
*Polydesmus complanatus*	5	2	2	–	–	–	1	1	–	–	11
*Polydesmus denticulatus*	9	7	8	1	4	–	2	4	7	3	45
*Polyxenus lagurus*	–	–	1	1	–	–	–	–	–	1	3
*Polyzonium germanicum*	–	–	–	–	–	–	–	1	–	–	1
*Trachysphaera acutula*	2	–	1	–	–	–	–	–	–	–	3
*Trachysphaera costata*	–	–	–	–	–	–	–	1	–	–	1
*Trachysphaera gibbula*	9	–	2	–	1	–	1	–	1	2	16
*Unciger foetidus*	3	4	2	1	–	–	1	2	2	–	15
*Unciger transsilvanicus*	–	–	1	–	1	–	3	–	–	–	5
Σ	105	66	68	35	59	29	57	47	53	46	565

### Effects of fixative solutions on diversity and activity of myriapods along the depth gradient of screes

At five of the study sites (sites 1–5; all situated in Slovakia), formaldehyde (two trap sets) and ethylene glycol (one trap set) were used in parallel. At all of these five study sites, much greater numbers of individuals and species of myriapods were recorded in subterranean traps with ethylene glycol than in the traps with formaldehyde; however, each of the systematic groups of Myriapoda responded specifically to the type of fixation.

**Figure 4.**
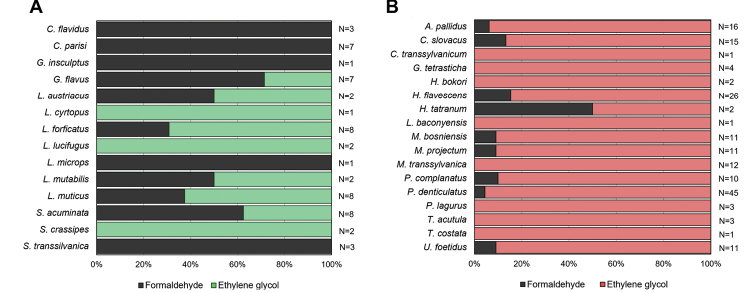
Graphical presentation of myriapod community characteristics in different fixative solutions (N = number of individuals). **A** Formaldehyde to ethylene glycol ratio of sampled centipede species from all study sites, where both fixating solutions were used **B** formaldehyde to ethylene glycol ratio of sampled millipede species from all study sites, where both fixating solutions were used.

**Figure 5.**
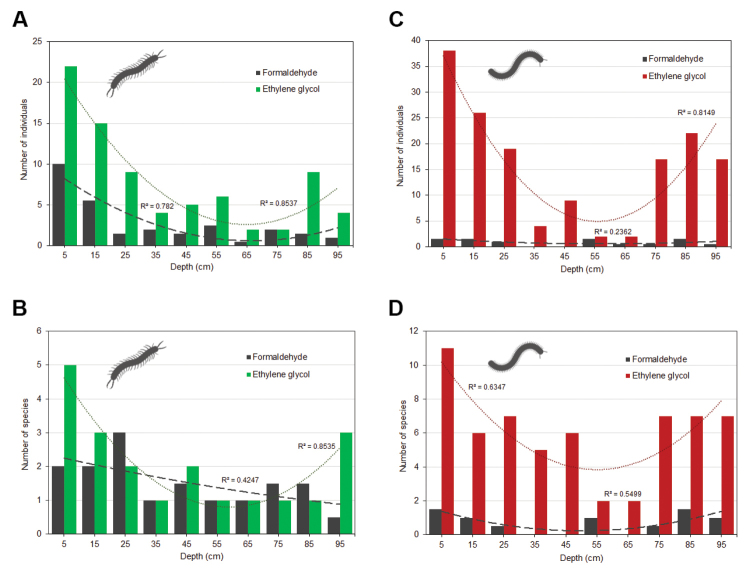
Vertical distribution of myriapods along the depth gradient in different fixative solutions (data recalculated for the same number of traps). Trend line: dashed = formaldehyde, dotted = ethylene glycol. **A** Vertical distribution of Chilopoda specimens along the depth gradient (5–95 cm) at five scree slopes in different fixative solutions **B** vertical distribution of centipede species along the depth gradient at five scree slopes in different fixative solutions **C** vertical distribution of Diplopoda specimens along the depth gradient (5–95 cm) at five scree slopes in different fixative solutions **D** vertical distribution of millipede species along the depth gradient at five scree slopes in different fixative solutions.

For centipedes, ethylene glycol appears to be more attractive (or less repellent) than formaldehyde. Higher numbers of individuals were collected in traps with ethylene glycol. However, regarding species composition, the effect of both fixatives seems to be complementary, as each of the solutions contained some species exclusive for particular fixative too (Fig. 4A). Vertical distribution of centipedes along the depth gradient at five scree slopes was characterised by strong decline and later small increase in deepest layers in the number of individuals in formaldehyde as well as ethylene glycol fixative solution, resembling its general U-shape curve of abundances (cf. Figs 2A, 5A). On the other hand, and contrary to ethylene glycol fixative, a simple decreasing trend in number of species was recorded in traps with formaldehyde fixative (Fig. 5B).

Regarding millipedes, ethylene glycol showed significantly higher activity and species diversity compared to formaldehyde. All collected species preferred traps with ethylene glycol; almost half of the millipede species occurred exclusively in this type of fixative solutions (Fig. 4B). Number of species and individuals decreased with depth in ethylene glycol fixation (down to 35 cm) and increased again at depths of 75–95 cm. Vertical distributions of millipedes in formaldehyde showed no particular trend (Fig. 5C). Similar patterns were recognized for number of species too (Fig. 5D).

## Discussion

Scree habitats serve as a prospective source of information on species habitat preference, diversity and potential migration between the shallow underground environment and the cave environment. Interspaces of forested scree slopes are inhabited by various groups of invertebrates, predominantly arthropods, represented by both edaphic and subterranean species (Juberthie 2000; Nitzu et al. 2014; Jiménez-Valverde et al. 2015). Our study confirmed that Central European myriapods colonise belowground zones in scree habitats covered with soil. It also indicates that there are some differences in diversity and distribution of centipede and millipede assemblages along depth profiles. The majority of myriapod species captured using subterranean traps represent common epigeic or edaphic fauna, occurring predominantly in the nutrient-rich uppermost layers of soil and litter. Some subterranean forms were captured, as well as species preferring scree slope habitats, including *Archiboreoiulus pallidus* and *Cibiniulus slovacus* (Antić et al. 2015; Mock et al 2015); these species are also cave-dwellers. However, some cave dwelling species known from the study region are absent in scree samples (Kováč et al. 2014).

The distribution pattern was characterised by typical sharp decline at the beginning of the measured depth gradient culminated near the middle zone, and increased occurrence and diversity of myriapods in the deeper parts of the gradient. Such decrease in abundance of the studied groups has been confirmed by several studies in different invertebrates (e.g., Laška et al. 2011; Rendoš et al. 2012, 2016a). Among other factors, the decline in upper layers may be due to the decreasing availability of organic resources as well as available spaces up to 50 cm (Gers 1998; Rendoš et al. 2012; Nitzu et al. 2014). On the other hand, from the depth of 70 cm we can see numerous communities of subterranean species that do not move to the upper layers. Some of the studies (Růžička et al. 1990; Růžička 1999; Laška et al. 2008) highlight the effect of substrate porosity, layer depth and vegetation on vertical distribution in soil. Regional surface species congregate deeper underground in screes similarly to caves especially in territories with rare obligate subterranean dwellers (Rendoš et al. 2016a; Fernandes et al. 2019).

The structure of centipede assemblage in scree habitats along the depth gradient has been described by various authors. In Querner and Gereben-Krenn (2005), the centipedes were distributed vertically only to a depth of 20 cm, while in our study, centipedes occurred at each depth of the gradient. Almost half of our collected centipedes were represented by *Lithobius forficatus*. The species is known for its wide ecological valency, characterised by Pan-European and Palearctic distribution. It has been documented from subterranean environment by Nitzu et al. (1999) from the caves of Romania (Ilie 2003a, 2003b) and caves of the Slovak Karst (Országh et al. 1994; Országh 2000). *Lithobius forficatus* is also known to penetrate deeper parts of the scree slope habitats (Rendoš et al. 2012, 2016a). The second-most abundant species of centipedes captured along the depth gradient was *Lithobius lucifugus*. This species was previously recorded at different karstic areas (e.g., Folkmanová 1951; Ilie 2003a, Ilie 2004) and is known to inhabit various underground habitats, including caves (Novák and Dányi 2011; Dvořák and Dvořáková 2015; Růžička et al. 2016; Tuf et al. 2017).

Relative abundance and diversity of millipedes captured using subterranean traps was higher in comparison to centipedes. Majority of the sampled species can be described as epigeic and edaphic. The most abundant species, collected only on the scree slopes of the Czech Republic, was *Ochogona caroli*. This species inhabits mainly higher altitudes of Central European mountains confirming its higher activity for the colder part of the year (Kocourek et al. 2017). Some important representatives of edaphic or subterranean forms were captured, including two species of family Blaniulidae, *Archiboreoiulus pallidus* and *Cibiniulus slovacus*; these species were captured in the deepest parts of depth gradient. Both species represent blind relict millipedes with similar ecological demands, known for their preference of shallow subterranean habitats. *Archiboreoiulus pallidus*, characterised by cryptic behaviour, has so far been documented only from the subterranean environment, for the studied area (Mock et al. 2015). Another blind relict species, *Cibiniulus slovacus*, was described only recently from caves in Slovakia (Antić et al. 2015); the species shows considerable affinity to forested scree habitats. The presence of similar species (morphotypes) that may coexist on a single site (e.g., chordeumatid diplopods of *Ochogona caroli* and *Listrocheritium septentrionale*) or that alternate similarly in the depth gradient (e.g., blind blaniulid millipedes and *Blaniulus guttulatus*, *Archiboreoiulus. pallidus*, and *Cibiniulus slovacus*) emphasises the need for precise taxonomic work. Another representative of subterranean millipede fauna captured in scree slopes is *Geoglomeris subterranea*. The species is morphologically well-adapted to subterranean environment and is known to inhabit various western-European caves (Gruber 1985; Kocourek et al. 2017). Its occurrence in soil has been documented (Wesener et al. 2019) even from artificial MSS habitat from the Czech Republic (Riedel et al. 2009). Apart from common European species, a few endemic species of millipedes were determined such as *Cibiniulus slovacus*, *Hungarosoma bokori*, *Hylebainosoma tatranum*, and *Listrocheritium septentrionale*. Some relict species with wider distribution include millipedes (*Geoglomeris subterranea*, *Archiboreoiulus pallidus*, *Trachysphaera* spp.) and centipedes (*Harpolithobius anodus*, *Lithobius cyrtopus*, *Lithobius forficatus*, *Lithobius nodulipes*) (Tuf and Tufová 2008; Kováč et al. 2014).

Nitzu et al. (2018a) emphasise, in addition to the geographical differences on the sites, the significant influence of the geological subsoil on the abundance and diversity of calciphile diplopods in favour of carbonate rocks. Although their study suggests a strong correlation of the calcareous geological substratum with an invertebrate species assemblage, our study found no statistically significant differences between assemblage abundance on limestone and non-limestone study sites either in Chilopoda or in Diplopoda (Chi-square test results).

Generalised additive models helped identify significant patterns of distribution, i.e., to find species, for which depth is useful predictor of its abundance. This analysis can record species with some preferences. A non-significant pattern of distribution could be evidence for either random distribution or equal distribution. Some species with equal depth distribution can be important and stable members of MSS, too. Such notable species in our material seem to be millipedes *Cibiniulus slovacus*, *Haasea flavescens*, *Ochogona caroli*, *Polydesmus denticulatus*, and *Trachysphaera gibbula*.

Five of the study sites used of two types of fixative solutions in parallel and brought different results. For millipedes, traps with ethylene glycol show much higher efficiency in comparison to those with formaldehyde. This is consistent with other studies referring to the effectiveness of fixative solutions on some invertebrates in forested scree slopes (Rendoš et al. 2014, 2016a; Mock et al. 2015; Rudy et al. 2018; Jureková et al. 2019). This phenomenon seems to be caused by the repellent effect of formaldehyde solution on some species, observed by several authors (e.g., Renner 1982; Gerlach et al. 2009; Rendoš et al. 2016a). However, some fixative solutions could serve as attractants for different species (Skvarla et al. 2014). Regarding species composition, each of the millipede species preferred ethylene glycol, with some species collected exclusively in this fixative. Altogether, for millipedes there is no benefit to using formaldehyde in comparison to ethylene glycol. Regarding millipedes, it seems there is no sense in using formaldehyde as a fixative. On the contrary, ethylene glycol has been proved to be a suitable fixation. As an inexpensive alternative to ethylene glycol, propylene glycol can be used (Skvarla et al 2014). Propylene glycol is practically non-toxic and has the same preservation properties as ethylene glycol (Jud and Schmidt-Engling 2008; Aristophanous 2010). In addition, it is suitable for molecular studies, as the quality of DNA conservation in undiluted propylene glycol seems to be similar to DNA preserved in ethanol (Moreau et al. 2013).

Positive effects of ethylene-glycol were also observed in centipedes, with higher abundance in ethylene glycol traps documented at majority of the study sites with two fixative liquids used. Species composition, however, showed selective effects of both fixative solutions, as some of the centipede species were collected only in formaldehyde and others only in ethylene glycol. An attractive effect of formaldehyde was observed only in case of symphylans, with more individuals collected in traps using this fixative solution. Any small change in traps, including using different fixatives, can affect results. In our study, we generalise the main features, yet each of the study sites has specific characteristics. For a long-term depth gradient study, it would be advisable to use a completely neutral fixative solution (water); however, this is in principle impossible. After a short period of time, in any type of solution, the carcasses of captured animals accumulate and become attractant or repellent for other animals. The types of subterranean traps used in this study have limitations; however, it is effective for comparison of results to other methods of collecting of soil fauna and does not require excessive and time-consuming effort.

## Conclusions

Subterranean diversity of Myriapoda inhabiting scree slopes has been investigated at various localities of mountainous Central Europe to the depth of one meter. Our study represents the first study with a larger number of sites dealing with the issue. Forested scree slopes in the region are usually lacking exclusively subterranean myriapod species and are largely colonised by surface-dwelling species of centipedes and millipedes. Deeper zones of screes are apparently parts of MSS and are clearly preferred only by two blind blaniulid millipedes, *Archiboreoiulus pallidus* and *Cibiniulus slovacus*. However, a relatively diverse community of relict myriapod fauna uses debris habitats as a climatic refugium. Although all studied locations and studied groups of invertebrates have their distinctive specifics, general depth distribution of Myriapoda has its pattern as well.

The design of subterranean traps had a significant effect on the findings. This was verified using two different fixative solutions in traps in parallel. The use of ethylene glycol in traps superimposed data obtained by formaldehyde; however, it did not provide a completely different picture of the depth distribution of myriapods. Any of the types of subterranean traps used in this study can only be recommended for any similar study.

## Supplementary materials

Supplementary material 1 Table S1 Data type Species data Explanation note List of sampled centipede species and community characteristics. Abbreviations: D-Dominance, C-Constancy. Coloured boxes indicate number of individuals in given depth. The numbers indicating particular studied scree slopes are stated in Fig. 1. https://binary.pensoft.net/file/404888This dataset is made available under the Open Database License (http://opendatacommons.org/licenses/odbl/1.0/). The Open Database License (ODbL) is a license agreement intended to allow users to freely share, modify, and use this Dataset while maintaining this same freedom for others, provided that the original source and author(s) are credited.Beáta Haľková, Ivan Hadrián Tuf, Karel Tajovský, Andrej Mock

Supplementary material 2 Table S2 Data type Species data Explanation note List of sampled millipede species and community characteristics. Abbreviations: D-Dominance, C-Constancy. Coloured boxes indicate number of individuals in given depth. The numbers indicating particular studied scree slopes are stated in Fig. 1. https://binary.pensoft.net/file/404889This dataset is made available under the Open Database License (http://opendatacommons.org/licenses/odbl/1.0/). The Open Database License (ODbL) is a license agreement intended to allow users to freely share, modify, and use this Dataset while maintaining this same freedom for others, provided that the original source and author(s) are credited.Beáta Haľková, Ivan Hadrián Tuf, Karel Tajovský, Andrej Mock
